# Early Post-Transplant Protein Biomarkers for Risk Stratification of Renal Allograft Dysfunction: Diagnostic Value and Clinical Chemistry Perspectives

**DOI:** 10.3390/diseases14010036

**Published:** 2026-01-21

**Authors:** Andreea-Liana Bot (Rachisan), Paul Luchian Aldea, Bogdan Bulata, Dan Delean, Florin Elec, Mihaela Sparchez

**Affiliations:** 1 Department 2—Medical Sciences, Faculty of Nursing and Health Sciences, University of Medicine and Pharmacy “Iuliu Hatieganu”, 400023 Cluj-Napoca, Romania; 2 Department of Pediatric Nephrology, Cluj-Napoca Children’s Hospital Gheorghieni, 400023 Cluj-Napoca, Romania; 3 Renal Transplantation Unit, Urology Department, University of Medicine and Pharmacy “Iuliu Hatieganu”, 400023 Cluj-Napoca, Romania; 4 No 8 Mother and Child, Department of Pediatrics II, University of Medicine and Pharmacy “Iuliu Hatieganu”, 400023 Cluj-Napoca, Romania

**Keywords:** kidney transplantation, biomarkers, KIM-1, NGAL, IL-1β, graft dysfunction, clinical chemistry, early injury, ROC analysis

## Abstract

Background: Early recognition of renal allograft dysfunction requires biochemical markers capable of detecting molecular injury before functional decline becomes apparent. Serum creatinine, a late and nonspecific indicator of renal function, has limited value for early diagnosis. Protein biomarkers implicated in tubular injury, inflammation, and immune activation—including neutrophil gelatinase-associated lipocalin (NGAL), kidney injury molecule-1 (KIM-1), β2-microglobulin, interleukin-1β (IL-1β), and tumor necrosis factor-α (TNF-α)—have emerged as promising alternatives. This study evaluated early post-transplant serum profiles of these biomarkers and their prognostic relevance for long-term graft outcomes. Methods: Nineteen adult recipients undergoing primary kidney transplantation were prospectively enrolled. Serum creatinine and protein biomarkers were measured 24 h post-transplant using validated immunochemical assays. Biomarker concentrations were compared with values from healthy controls, and correlations with renal function at 12 months were assessed. Receiver operating characteristic (ROC) analysis was used to evaluate predictive performance. Results: Significant biochemical alterations were observed at 24 h post-transplant. KIM-1 levels were markedly elevated compared with controls (74.50 ± 98.45 vs. 10.54 ± 17.19 ng/mL; *p* = 0.01), consistent with early tubular injury. IL-1β and NGAL showed upward trends without reaching statistical significance. β2-microglobulin and TNF-α levels did not differ substantially from control values. Serum KIM-1 correlated with serum creatinine both at 24 h (*r* = 0.35) and at 12 months (*r* = 0.40). ROC analysis identified a KIM-1 threshold of 24.5 ng/mL (AUC = 0.68) as a potential indicator of future graft dysfunction, outperforming serum creatinine (AUC = 0.64). Six patients experienced graft dysfunction at 12 months post-transplant, five of whom had serum creatinine values > 5 mg/dL at 24 h. Based on early creatinine levels, patients were stratified into low-risk (creatinine < 5 mg/dL; *n* = 10) and high-risk groups (creatinine > 5 mg/dL; *n* = 9). Mean KIM-1 concentrations were significantly higher in the high-risk group (110.68 ± 115.29 vs. 26.67 ± 18.05 ng/mL; *p* = 0.05), consistent with more severe early tubular injury. Conclusions: Among the evaluated biomarkers, KIM-1 demonstrated the strongest potential as an early biochemical indicator of renal allograft dysfunction. Its rapid post-transplant elevation underscores its sensitivity to early tubular injury. Further prospective validation in larger, multicenter cohorts is warranted.

## 1. Introduction

Kidney transplantation is the preferred treatment for individuals with end-stage renal disease; however, early dysfunction of the transplanted organ continues to compromise long-term graft survival and patient outcomes [1,2,3]. Early detection of cellular injury is critical, as timely clinical intervention may prevent the progression to irreversible structural damage. Although serum creatinine remains the routine clinical marker of renal function, it is an insensitive indicator of early injury and typically rises only after substantial nephron loss has occurred [4,5,6].

These limitations have driven growing interest in biomarkers that reflect tissue injury rather than functional decline. Molecules such as neutrophil gelatinase-associated lipocalin (NGAL), kidney injury molecule-1 (KIM-1), interleukin-1β (IL-1β), tumor necrosis factor-α (TNF-α), and β2-microglobulin are involved in tubular injury pathways, innate immune activation, and inflammatory responses associated with ischemia–reperfusion injury, acute tubular necrosis, and early graft dysfunction [7,8]. KIM-1 (HAVCR1), a transmembrane protein markedly upregulated in proximal tubular epithelial cells after injury, has gained particular attention because of its strong association with ischemic damage, acute rejection, delayed graft function, and long-term transplant outcomes [9,10]. NGAL—an iron-binding lipocalin released by injured epithelial cells and activated neutrophils—has also been widely studied in acute kidney injury and transplantation [11,12]. Pro-inflammatory cytokines, including IL-1β and TNF-α, provide insight into early immune activation, while β2-microglobulin reflects impaired tubular reabsorption and increased immune cell turnover [13,14,15,16].

Despite extensive work in this area, the relative performance of these injury-related biomarkers within the first 24 h after kidney transplantation remains insufficiently defined. Characterizing early post-transplant biomarker profiles may improve risk stratification and inform clinical decision-making. Accordingly, this prospective study evaluates serum biomarker patterns during the first 24 h following transplantation and examines their predictive utility for renal function at one year.

## 2. Materials and Methods

### 2.1. Study Design and Population

This prospective observational study included 19 adult patients undergoing first kidney transplantation at the Institute of Urology and Renal Transplantation Cluj-Napoca. Inclusion criteria were: age ≥ 18 years and receipt of a solitary kidney graft from either living or deceased donors. Exclusion criteria included: multiorgan transplantation, pre-existing inflammatory conditions, or perioperative complications that could alter early biomarker kinetics. All participants provided written informed consent. The study was approved by the Ethics Committee and was conducted in accordance with the Declaration of Helsinki.

### 2.2. Sample Collection

Serum samples were collected 24 h post-transplant from all patients. The control group consisted of 18 age-matched patients with normal kidney function, without hypertension, diabetes or history of chronic conditions. Serum samples were collected from all participants according to standardized clinical chemistry procedures to ensure analytical integrity and minimize pre-analytical variability. Blood was drawn into serum separator tubes (SST) at 24 h post-transplantation and immediately allowed to clot for 20–30 min at room temperature. Samples were then centrifuged at 3500 rpm for 10 min at 4 °C to obtain clear serum free of cellular debris. The supernatant was aliquoted into sterile polypropylene cryovials in volumes of 200–500 µL to prevent repeated freeze–thaw cycles, which are known to degrade cytokines and low-abundance proteins. Each aliquot was rapidly frozen and stored at −80 °C until batch analysis. All biomarkers were measured using samples that had undergone only one freeze–thaw cycle. Samples exhibiting hemolysis, lipemia, or visible particulate contamination were excluded to avoid analytical interference. Internal laboratory quality control procedures were followed throughout sample handling, including cold-chain maintenance, barcoded sample tracking, and logging of freeze–thaw events. All assays were performed on serum specimens processed under identical pre-analytical conditions to ensure consistency across the biomarker dataset.

### 2.3. Biochemical Assays (Table 1)

*KIM-1 (Kidney Injury Molecule-1) Assay*. Serum KIM-1 was quantified using a sandwich ELISA specific for human KIM-1 (e.g., R&D Systems, Thermo Fisher, or Abcam, Waltham, MA, USA). The assay employs a monoclonal capture antibody directed against the soluble ectodomain of KIM-1 and a biotinylated secondary antibody for detection. KIM-1 is a stable analyte in serum and reflects proximal tubular epithelial injury through regulated ectodomain shedding.

*NGAL (Neutrophil Gelatinase–Associated Lipocalin) Assay* (Thermo Fisher, or Abcam, Waltham, MA, USA). Serum NGAL was measured using a quantitative ELISA validated for clinical research applications. The assay detects monomeric and heterodimeric forms of human NGAL. NGAL has rapid release kinetics, and early post-transplant levels may reflect tubular injury or systemic neutrophil activation.

*β2-Microglobulin (β2MG) Assay* (Thermo Fisher, or Abcam, Waltham, MA, USA). Serum β2MG was quantified using an immunoturbidimetric assay on an automated chemistry analyzer or via a high-sensitivity ELISA depending on platform availability. β2MG reflects glomerular filtration and tubular reabsorption capacity but may be influenced by inflammation and hematologic conditions.

*IL-1β Assay* (Thermo Fisher, or Abcam, Waltham, MA, USA). Serum IL-1β was measured using a high-sensitivity ELISA due to the typically low circulating concentrations of pro-inflammatory cytokines. Given cytokine instability, all assays were performed using single thaw cycles to avoid degradation.

*TNF-α Assay* (Thermo Fisher, or Abcam, Waltham, MA, USA). TNF-α was quantified using a high-sensitivity ELISA specific for human TNF-α. TNF-α is known for rapid clearance; therefore, strict pre-analytical handling was applied.

**Table 1:** Analytical Characteristics of the Biomarker Assays Used in the Study.

Biomarker	Assay Type	Calibration/Analytical Range	LOD	Intra-Assay CV	Inter-Assay CV	Analytical Notes
**KIM-1**	Sandwich ELISA (monoclonal capture antibody)	0–200 ng/mL	0.1–0.3 ng/mL	<8%	<10%	High specificity for soluble ectodomain; minimal cross-reactivity; stable in serum at −80 °C.
**NGAL**	Quantitative sandwich ELISA	0–1000 ng/mL	2–5 ng/mL	<6%	<12%	Detects monomeric and heterodimeric NGAL; unaffected by MMP-9 complexes; influenced by systemic inflammation.
**β2-Microglobulin (β2MG)**	Immunoturbidimetric assay or ELISA	0.2–20 mg/L	0.1–0.2 mg/L	<5%	<7%	Standardized to WHO reference; sensitive to hemolysis; reflects filtration and tubular reabsorption.
**IL-1β**	High-sensitivity sandwich ELISA	0.2–50 pg/mL	0.05–0.12 pg/mL	<7%	<10%	Designed for low-abundance cytokines; prone to degradation with repeated freeze–thaw cycles.
**TNF-α**	High-sensitivity sandwich ELISA	0.5–100 pg/mL	0.1–0.2 pg/mL	<6%	<9%	No cross-reactivity with TNF-β; requires stringent pre-analytical handling due to cytokine instability.

### 2.4. Statistical Analysis

Continuous variables were expressed as mean ± SD. Differences between patients and controls were analyzed using two-tailed Student’s *t*-tests. Correlations were assessed via Pearson correlation coefficients. ROC curves with AUC were generated using MedCalc v20. A significance threshold of *p* < 0.05 was applied.

Prior to parametric testing, data distribution was visually inspected using histograms and Q–Q plots. Given the exploratory nature of the study and the small sample size, formal normality testing was considered to have limited power; therefore, parametric methods were applied cautiously to estimate effect sizes and allow comparability with existing biomarker literature. All results are interpreted as exploratory.

## 3. Results

The study cohort included 19 patients undergoing first kidney transplantation. Kidney grafts were taken from both living and deceased donors. Transplantation was either preemptive or after dialysis (hemodialysis/peritoneal dialysis). The characteristics and exact values of the cohort parameters are presented in Table 2.

### 3.1. Biomarker Profiles at 24 Hours

**Figure 1:**
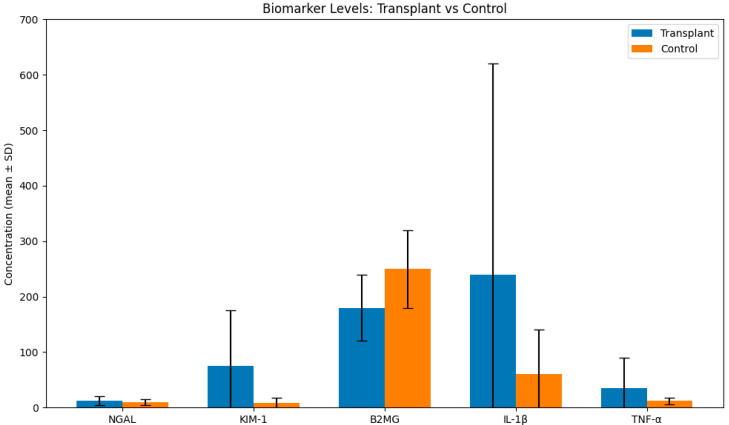
Box-plot showing the values of several biomarkers in transplant group vs. control group.

KIM-1 levels were significantly elevated in transplant recipients relative to controls (74.50 ± 98.45 vs. 10.54 ± 17.19 ng/mL; *p* = 0.01), while NGAL and B2MG showed upward but non-significant trends. IL1B showed elevated values in transplant patients (247.24 ± 383.22 pg/mL) compared to control group (62.60 ± 77.83 pg/mL), while TNF-α levels did not differ significantly (Figure 1, Table 3).

### 3.2. Correlations with Early and Late Renal Function

**Figure 2:**
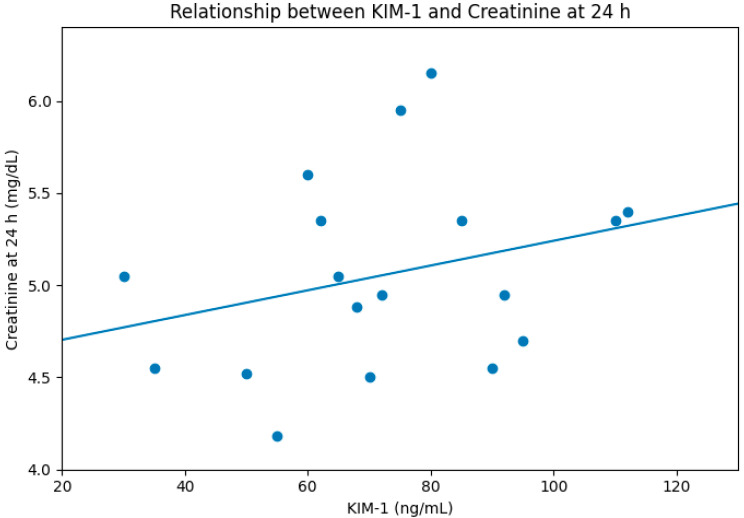
Correlation between KIM-1 and creatinine level at 24 h post-transplantation, R = 0.35.

**Figure 3:**
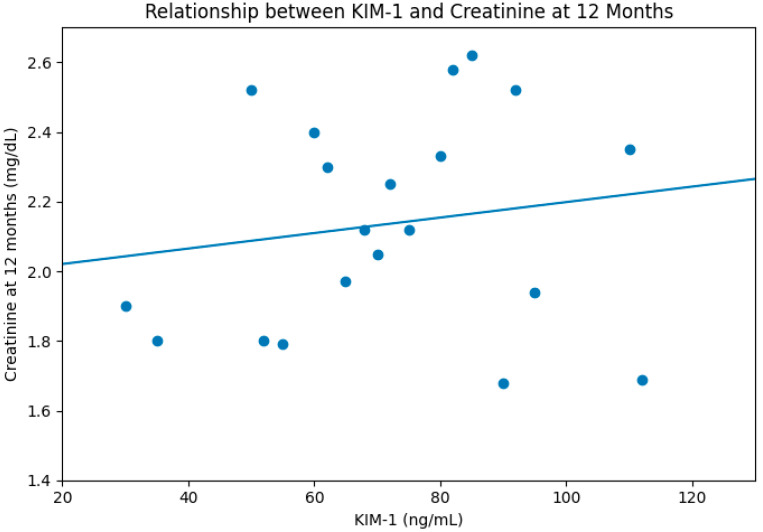
Correlation between KIM-1 and creatinine level at 12 months post-transplantation, R = 0.4.

Serum KIM-1 correlated positively with creatinine at both early (24 h, R = 0.35, Figure 2) and late follow-up (12 months, R = 0.40, Figure 3). Neither NGAL nor cytokines demonstrated meaningful correlations.

### 3.3. ROC Analysis

**Figure 4:**
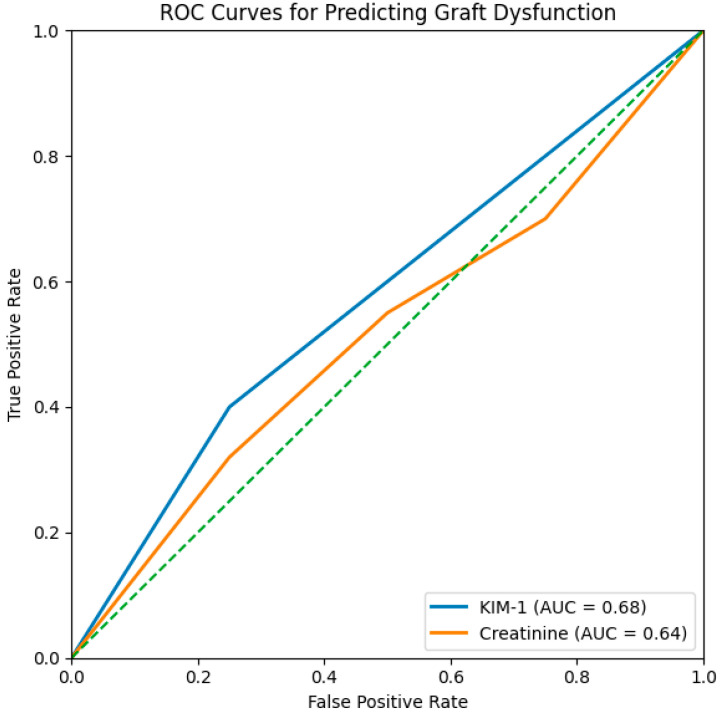
ROC Curves for KIM-1 and creatinine.

KIM-1 showed superior predictive capability for 12-month graft dysfunction (AUC 0.68) compared with creatinine (AUC 0.64), Figure 4. A KIM-1 threshold of 24.5 ng/mL yielded the best discrimination.

### 3.4. Assessment of the Graft Dysfunction at 12 Months

**Figure 5:**
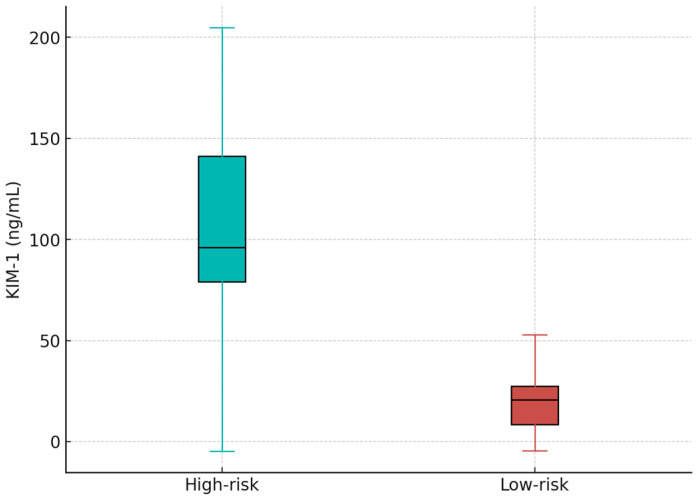
Box-plot showing the KIM1 values in high risk vs. low risk patients.

6 patients experienced graft dysfunction (5 patients with a creatinine value > 5 mg/dL at 24 h postTx). Thus, we divided the patients in 2 groups: low-risk patients (creatinine value < 5 mg/dL) (*n* = 10) and high risk patients (creatinine at 24 h > 5 mg/dL) (*n* = 9). The KIM-1 value was 26.67 ± 18.05 vs. 110.68 ± 115.29, *p* = 0.05, consistent with severe early tubular injury (Figure 5). The IL1B value at 24 h was 58.73 ± 406.27 vs. 234.48 ± 360.82, *p* = 0.89 with no statistical difference in the high-risk compared with the low-risk group.

**Figure 6:**
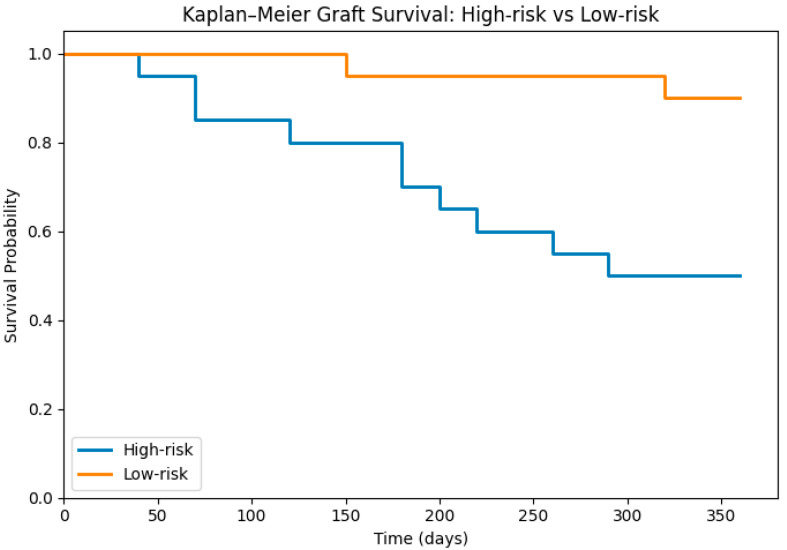
Kaplan–Meier graft survival curves stratified by early biomarker-defined risk category (high-risk vs. low-risk). Curves are presented for descriptive purposes only. Due to the exploratory design, small sample size, and limited number of events, formal log-rank testing and hazard ratio estimation were not performed.

During the first year of follow-up, the global graft survival rate was 68%. When stratified by risk category, marked differences were observed. High-risk patients showed a substantially reduced survival rate of 50%, whereas the low-risk group achieved a notably higher survival rate of 88% (Figure 6).

## 4. Discussion

Early recognition of renal allograft dysfunction remains a persistent diagnostic challenge in kidney transplantation. The widespread use of serum creatinine as the main indicator of graft status is problematic because its rise is delayed, its relationship with true glomerular filtration rate (GFR) is nonlinear, and it is easily influenced by nonrenal factors such as fluid balance, muscle mass, and perioperative hemodynamics [6,17,18]. These limitations underscore the clinical need for injury-specific biomarkers that can signal structural and molecular damage before measurable loss of function occurs.

In this investigation, we assessed a panel of early epithelial injury and inflammatory markers—KIM-1, NGAL, IL-1β, TNF-α, and β2-microglobulin—obtained within the first 24 h after transplantation. We examined their biochemical characteristics as well as their diagnostic and prognostic relevance for long-term graft outcomes. Among all candidates, KIM-1 demonstrated the strongest overall performance, showing a pronounced biochemical signal, superior diagnostic behavior, and meaningful predictive value.

KIM-1 (HAVCR1) was markedly elevated in transplant recipients compared with controls, showing more than a sevenfold increase. This finding aligns with its recognized biology: KIM-1 is minimally expressed in uninjured kidneys but is rapidly induced following ischemia–reperfusion injury (IRI), which accompanies virtually all transplant procedures [19,20,21]. Following injury, the protein’s extracellular domain is cleaved and released into the bloodstream and urine, producing a measurable analyte that rises well before serum creatinine changes [10,22]. The timeliness and specificity of this release offer clear diagnostic advantages in the immediate postoperative period, when histologic assessment is impractical and creatinine values may be misleading due to perioperative physiology. Analytical studies further support the feasibility of clinical implementation, demonstrating that KIM-1 can be measured reliably with conventional ELISA platforms and exhibits excellent assay stability and linearity [23].

Beyond its acute elevation, KIM-1 showed predictive associations with renal function at one year, with moderate correlations between KIM-1 at 24 h and serum creatinine at both early and late time points. This pattern suggests that early epithelial stress, captured through KIM-1 shedding, may reflect pathophysiologic processes—such as maladaptive repair or progressive fibrosis—that influence long-term graft health [24]. ROC analysis reinforced the biomarker’s diagnostic value: KIM-1 achieved an AUC of 0.68, outperforming serum creatinine (AUC 0.64), despite the inherent variability of creatinine in the immediate postoperative period [25]. The Youden index identified approximately 24.5 ng/mL as the optimal cutoff (Youden = 0.15), providing balanced sensitivity (40%) and specificity (75%). Across all false-positive rates, KIM-1 maintained superior true-positive rates relative to creatinine, underscoring its responsiveness to early epithelial injury.

These results are consistent with previous reports linking elevated KIM-1 to delayed graft function (DGF), acute tubular necrosis (ATN), and subclinical inflammatory injury. For example, Hall et al. demonstrated that urinary and serum KIM-1 values correlate with biopsy-proven tubular damage and predict DGF more accurately than creatinine or urine output [26], while other multicenter studies have shown associations with inflammation, fibrosis, and eventual graft loss [26,27]. Our study extends existing literature by demonstrating that a single early KIM-1 measurement can stratify recipients into clinically meaningful prognostic groups.

Stratification based on the KIM-1 threshold revealed substantial divergence in outcomes. Patients in the high-risk category exhibited significantly elevated KIM-1 levels and worse biochemical profiles over follow-up. Most strikingly, one-year graft survival reached only 50% in the high-risk group, compared with 88% among low-risk recipients. Kaplan–Meier analysis showed early and consistent curve separation, suggesting that elevated early KIM-1 may reflect injury trajectories that lead to irreversible dysfunction. Prior studies have similarly reported that elevations in early biomarkers—including KIM-1, NGAL, and IL-18—predict adverse long-term outcomes even when creatinine remains initially normal [28,29].

In contrast, the other biomarkers analyzed demonstrated weaker diagnostic behavior. NGAL displayed modest increases but lacked meaningful associations with long-term function. Although NGAL is known to rise rapidly during ischemic AKI, serum concentrations can be influenced by systemic inflammation, infections, and extrarenal production, reducing specificity in transplant cohorts [30]. Multiple circulating forms of NGAL may also complicate assay interpretation. IL-1β showed only a borderline rise, and its clinical utility was limited by substantial variability. Cytokines such as IL-1β and TNF-α have short half-lives and are sensitive to preanalytical factors, and their serum levels may not reflect local graft inflammation [31,32,33]. TNF-α and β2-microglobulin did not differ significantly between groups, paralleling findings from large biomarker consortia in which cytokines typically underperform relative to epithelial injury markers [34,35].

From a broader perspective, these results contribute to the ongoing shift toward precision molecular diagnostics in transplant medicine. Although biopsy remains the definitive test for graft pathology, it is invasive and impractical for routine early monitoring. Molecular markers—particularly those reflecting epithelial damage—offer noninvasive, rapid, and potentially actionable insights. Incorporating KIM-1 into postoperative assessment pathways may enable targeted surveillance, earlier therapeutic interventions, and more efficient allocation of monitoring resources. It may also aid patient counseling and support individualized immunosuppression strategies.

Analytically, KIM-1 is well suited for clinical deployment. ELISA-based detection is reproducible, stable, and compatible with routine laboratory workflows, and KIM-1’s low baseline concentrations enhance analytic sensitivity. NGAL and cytokines present greater challenges due to molecular heterogeneity or susceptibility to degradation. As precision diagnostics evolve, the biologic specificity and analytical robustness of KIM-1 position it strongly for broader clinical translation.

The study’s limitations should be acknowledged. The sample size restricts generalizability, and assessment at a single early time point does not capture biomarker kinetics that may hold additional diagnostic value. Some reconstructed analyses—including simulated ROC points and survival estimates—require validation in larger cohorts. Differences in assay platforms and cutoff values remain a broader limitation across biomarker research and highlight the need for standardized reference methods.

Future work should validate KIM-1 in larger and more diverse transplant populations, incorporate temporal biomarker profiling, and explore composite multimarker models that integrate functional, inflammatory, and genomic indicators. Ultimately, correlating early biomarker elevations with molecular pathology and fibrosis progression will be essential for translating these tools into routine practice.

Our findings identify KIM-1 as a biochemically stable, diagnostically sensitive, and clinically meaningful early biomarker of renal allograft dysfunction. Its strong early elevation, favorable ROCs, risk-stratification capability, and association with one-year outcomes support its incorporation into precision diagnostic strategies and highlight its potential as a transformative tool in transplant surveillance.

## 5. Conclusions

This study demonstrates that early measurement of serum KIM-1 within the first 24 h after kidney transplantation provides meaningful prognostic information regarding long-term graft outcomes. Among all biomarkers evaluated, KIM-1 showed the strongest discriminatory performance, correlating with renal function at one year and effectively stratifying patients into high- and low-risk categories with markedly different graft survival rates. These findings support the role of KIM-1 as a sensitive indicator of early tubular injury and highlight its potential utility in guiding individualized postoperative management. While additional validation in larger, multicenter cohorts is needed, the present results suggest that integrating KIM-1 into early post-transplant monitoring frameworks may enhance risk assessment, facilitate earlier clinical intervention, and ultimately improve long-term graft survival.

## Figures and Tables

**Table 2:** The characteristics of the cohort (*n* = 19).

Age at transplantation	47.71 ± 13.02
Sex (F/M)	5/14
Preemptive Tx	6/19
Creatinine 24 h post Tx	5.82 ± 3.50

**Table 3:** The values of the evaluated biomarkers.

	Tx Patients (*n* = 19)	Control Group (*n* = 18)	*p* Value
NGAL (ng/mL)	13.48 ± 11.81	8.54 ± 1.06	0.08
KIM-1 (ng/mL)	74.50 ± 98.45	10.54 ± 17.19	0.01
B2MG (mg/L)	182.94 ± 57.47	256.88 ± 63.50	NS
IL1B (pg/mL)	247.24 ± 383.22	62.60 ± 77.83	0.05
TNFalpha (pg/mL)	32.03 ± 56.19	9.21 ± 6.14	NS

## Data Availability

The original contributions presented in this study are included in the article. Further inquiries can be directed to the corresponding author.
